# Predictive Biomarkers for Response to Immunotherapy in Triple Negative Breast Cancer: Promises and Challenges

**DOI:** 10.3390/jcm12030953

**Published:** 2023-01-26

**Authors:** Xiaoxiao Wang, Laetitia Collet, Mattia Rediti, Véronique Debien, Alex De Caluwé, David Venet, Emanuela Romano, Françoise Rothé, Christos Sotiriou, Laurence Buisseret

**Affiliations:** 1 Breast Cancer Translational Research Laboratory J.-C. Heuson, Institut Jules Bordet, Hôpital Universitaire de Bruxelles (H.U.B), Université Libre de Bruxelles (ULB), 1000 Brussels, Belgium; 2 Academic Trials Promoting Team, Institut Jules Bordet, Hôpital Universitaire de Bruxelles (H.U.B), Université Libre de Bruxelles (ULB), 1000 Brussels, Belgium; 3 Radiotherapy Department, Institut Jules Bordet, Hôpital Universitaire de Bruxelles (H.U.B), Université Libre de Bruxelles (ULB), 1000 Brussels, Belgium; 4 Centre for Cancer Immunotherapy, Oncology Department, INSERM U932, Institut Curie, PSL Research University, 75005 Paris, France; 5 Medical Oncology Department, Institut Jules Bordet, Hôpital Universitaire de Bruxelles (H.U.B), Université Libre de Bruxelles (ULB), 1000 Brussels, Belgium

**Keywords:** triple negative breast cancer, immunotherapy, biomarker, tumor heterogeneity, molecular classification

## Abstract

Triple negative breast cancer (TNBC) is a highly heterogeneous disease with a poor prognosis and a paucity of therapeutic options. In recent years, immunotherapy has emerged as a new treatment option for patients with TNBC. However, this therapeutic evolution is paralleled by a growing need for biomarkers which allow for a better selection of patients who are most likely to benefit from this immune checkpoint inhibitor (ICI)-based regimen. These biomarkers will not only facilitate a better optimization of treatment strategies, but they will also avoid unnecessary side effects in non-responders, and limit the increasing financial toxicity linked to the use of these agents. Huge efforts have been deployed to identify predictive biomarkers for the ICI, but until now, the fruits of this labor remained largely unsatisfactory. Among clinically validated biomarkers, only programmed death-ligand 1 protein (PD-L1) expression has been prospectively assessed in TNBC trials. In addition to this, microsatellite instability and a high tumor mutational burden are approved as tumor agnostic biomarkers, but only a small percentage of TNBC fits this category. Furthermore, TNBC should no longer be approached as a single biological entity, but rather as a complex disease with different molecular, clinicopathological, and tumor microenvironment subgroups. This review provides an overview of the validated and evolving predictive biomarkers for a response to ICI in TNBC.

## 1. Introduction

Triple negative breast cancer (TNBC) represents 15 to 20% of all breast cancers (BC) and is characterized by the lack of hormone receptor (HR) and human epidermal growth factor receptor (HER2) expression. Compared to other BC subtypes, TNBC is associated with a dismal prognosis. Recently, combinations of immune checkpoint inhibitors (ICIs) targeting the programmed death-1 (PD-1) protein or its ligand (PD-L1) and chemotherapy were shown to significantly improve the clinical outcome of patients with TNBC, both in the early (eTNBC) and in the metastatic (mTNBC) setting [1,2,3,4,5,6,7]. Nevertheless, while meaningful and durable responses were achieved in some TNBC patients, the majority did not benefit from this treatment. Research efforts are therefore ongoing to identify reliable predictive biomarkers. This will increase the efficiency of ICIs, and avoid toxicities and unnecessary costs in non-responders [8].

However, apart from germline *BRCA1/2* mutations to select patients that may benefit from a treatment with poly ADP ribose polymerase (PARP) inhibitors, TNBC is treated as a single disease entity. Nevertheless, TNBC encompasses a variety of histologic types and genomic alterations, and has been subdivided in specific molecular subtypes with distinct tumor characteristics and clinical outcomes [8,9]. This heterogeneity contributes to differences in the prognosis and responses to therapies in patients with TNBC.

TNBC molecular subtypes were established based on gene expression profiling and further characterized by evaluating the mutational, copy number, epigenetic, proteomic, and phospho-proteomic patterns [9,10,11,12]. Lehmann et al. initially depicted six molecular subtypes including basal-like 1 and 2 (BL1 and BL2), immunomodulatory (IM), mesenchymal (M), mesenchymal stem-like (MSL), and luminal androgen receptor (LAR), which were later amended into four subtypes (BL1, BL2, M, and LAR) [10,13]. Other groups–including ours–have further refined this classification to better capture the TNBC heterogeneity [11,14]. 

Recent studies demonstrated that different TNBC molecular subtypes are associated with distinct tumor microenvironment (TME) features, including specific immune infiltrates and the expression of distinct, targetable immune pathways [9,15], indicating that some TNBC subtypes may be more suitable candidates for immunotherapeutic strategies. Although PD-L1 immunohistochemistry (IHC) expression is not an optimal biomarker, it has been shown to be predictive for the response to the combination of a PD-1/PD-L1 ICI with chemotherapy in patients with metastatic mTNBC, but not when these regimens are being used as a neoadjuvant treatment in eTNBC [1,2,3,6,7,16]. This inconsistency could be related to the dynamics of PD-L1 expression during treatment, which are influenced by several mechanisms including genomic alterations, epigenetic modifiers, and transcriptional regulation. In addition to this, specific features of the TME, which differ across organs and disease stages, seem to have an influence on PD-L1 expression. Microsatellite instability (MSI) and high tumor mutational burden (TMB-H) are “agnostic” biomarkers approved for the use of the ICI in solid tumors [17,18]. However, both MSI and TMB-H are rare in TNBC and, as such, these biomarkers would only identify a small proportion of TNBC patients that are eligible for ICI therapy [19,20,21,22]. 

**Figure 1:**
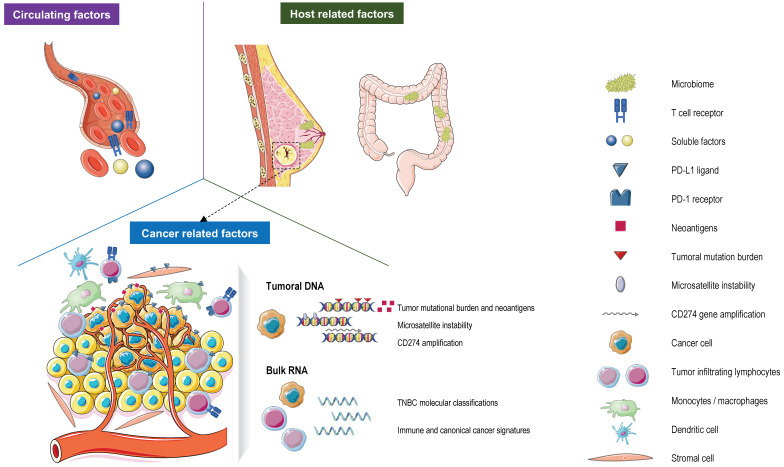
Currently validated and evolving predictive biomarkers for response to immune checkpoint inhibitors (ICIs) in patients with TNBC. Abbreviations. PD-L1: programmed death-ligand protein 1; PD-1: programmed death-1.

This article will review the available data on clinically approved and potential biomarker candidates for the response to ICI therapy in TNBC. To this end, we first evaluate prospective and retrospective studies including patients treated with ICIs in monotherapy or in combination with chemotherapy (Figure 1; Table 1). Subsequently, we interrogate the impact of TNBC heterogeneity on ICI sensitivity considering the different TNBC molecular subtypes. Finally, we discuss the complexity of TNBC and assess how this may impact the future assessment of ICI biomarkers in this setting.

## 2. Deciphering TNBC Heterogeneity with Molecular Classifications

In the past decade, large-scale sequencing studies attempted to classify TNBC based on somatic genomic alterations, which led to the identification of mutations associated with specific clinical outcomes and therapeutic responses [12,23]. Meanwhile, transcriptome profiling analyses from bulk RNA sequencing (RNA-seq) provided more robust insights into the heterogeneity of TNBC. The first gene expression-based TNBC classification was reported by Lehmann et al. in 2011 and described 6 molecular subtypes: BL1, BL2, IM, M, MSL, and LAR. The authors aligned representative TNBC cell lines to each of these 6 TNBC subtypes, revealing distinct therapeutic vulnerabilities to several agents [10]. This classification was later refined to include 4 molecular subtypes (BL1, BL2, M, and LAR), excluding the IM and MSL subtypes as these signatures proved to be mainly driven by lymphocytes and stroma cells [13]. More recently, our research group refined the Lehmann TNBC-6 type classification into 5 groups (BL, M, LAR, MSL, and IM) by removing the molecularly unstable BL2 subtype [14]. By combining gene expression profiling and copy number variations (CNVs), Burstein et al. proposed 4 distinct TNBC subtypes referred to as LAR, Mesenchymal (MES), basal-like immune-suppressed (BLIS), and basal-like immune-activated (BLIA) tumors [11]. Among these groups, the IM and BLIA-related subtypes are characterized by a higher expression of immune gene signatures and potentially targetable immune checkpoints, and are associated with a better prognosis [10,13,14,24]. The BL subtype in the Bareche et al. classification has an intermediate prognosis and is characterized by genomic instability, with DNA repair gene deficiency, and a high rate of *TP53* mutations. In contrast, M and MSL tumors are mainly associated with angiogenesis and stroma signatures. Finally, the LAR subtype, which is characterized by androgen receptor (AR) expression, is usually associated with a worse prognosis and is enriched for *PIK3CA*, *AKT1*, and *CDH1* mutations [14]. 

**Figure 2:**
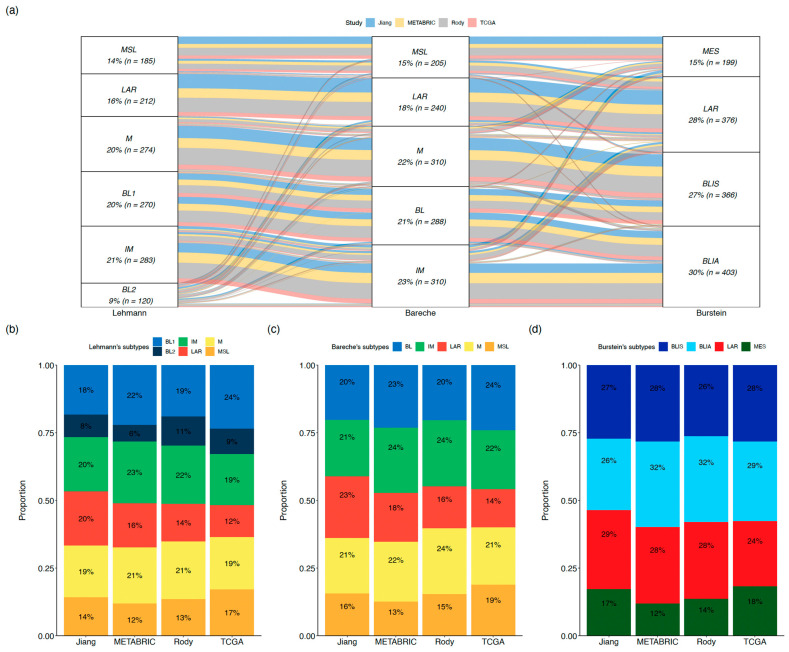
Lehmann’s TNBC type-6, Bareche’s, and Burstein’s molecular classifications across public datasets. (**a**) Overlap of TNBC molecular subtypes across public datasets (**b**–**d**). Distribution of Lehmann’s TNBC type-6, Bareche’s subtypes, and Burstein’s subtypes across each public dataset. Abbreviations. BL: basal-like, BLIA: basal-like immune-activated, BLIS: basal-like immune-suppressed, IM: immunomodulatory, LAR: luminal androgen receptor, M: mesenchymal, MES: mesenchymal, MSL: mesenchymal stem-like.

By comparing the molecular subtypes of 1,344 TNBC tumors from public datasets (Molecular Taxonomy of Breast Cancer International Consortium (METABRIC), The Cancer Genome Atlas Consortium (TCGA), Rody et al. and Jiang et al.) using 3 classification systems (Lehmann et al., Bareche et al. and Burstein et al.), we observed consistency across molecular subtypes from different classification systems depicting similar phenotypes, although some discordances were also observed [10,11,12,14,25,26,27] (Figure 2a). Among the three classification systems, the proportion of each molecular subtype and its contribution to TNBC heterogeneity are reproducible across the four datasets (Figure 2b–d, Appendix A).

Responses to ICI-based therapies in the function of the TNBC subtypes have been retrospectively evaluated in several clinical trials. In the phase I PCD4989 g trial, including a mTNBC cohort treated with atezolizumab monotherapy, the BLIA and LAR subtypes, characterized by higher levels of immune biomarkers (tumor infiltrating lymphocytes (TILs), PD-L1, and CD8 IHC expression) compared to M and BLIS tumors according to Burstein, benefitted most from atezolizumab [28]. In the IMpassion 130 trial in mTNBC, a retrospective analysis using Burstein’s classification revealed an improved outcome with atezolizumab and chemotherapy as the first-line treatment in the BLIA subtype, whereas LAR tumors, enriched for angiogenesis/estrogen receptor (ER) response pathways, seemed to be resistant to ICIs. Of note, in the PD-L1-negative subgroup, the BLIA subtype lost its predictive value, highlighting the need to consider the interdependency between a biomarker and the molecular subtype [29]. 

In the neoadjuvant NeoTRIPaPDL1 trial, a different molecular classification, namely “TNBCtypes”, was defined by applying a 101-gene algorithm that did not include the IM component [30]. In this study, pre-treatment TNBCtypes were not predictive for a benefit from atezolizumab, although a non-significant trend showed a higher pathologic complete response (pCR) rate (70%) in patients with BL1 tumors in the atezolizumab plus the chemotherapy arm compared to chemotherapy alone (54%). The pCR rate of the LAR subtype was low in both arms (22% and 19% for chemotherapy with and without atezolizumab, respectively) [32]. Interestingly, patients with a M subtype tumor displayed high pCR rates irrespective of whether they received atezolizumab, or not (60% and 50%, respectively; difference not statistically significant) [32]. Interestingly, on-treatment TNBCtypes assessed at day 1 of cycle 2 proved to be predictive for pCR in this study (*p* = 0.00002). In fact, compared to BL1 tumors, LAR and M tumors were associated with a significantly lower pCR rate in both treatment arms, irrespective of the PD-L1 expression and stromal TILs levels [33]. In light of these results, the IM and BLIA-related subtypes seem to be the most suitable candidates for immunotherapy. This is consistent with previous findings demonstrating that immune-related molecular subtypes are enriched in tumors which are infiltrated by TILs and express PD-L1 [15].

Interestingly, data show that the TNBC molecular subtypes can evolve over time and can differ between pre- and post-treatment. For example, according to the Lehmann et al. classification, the most frequent change was from BL1 to M subtypes (38%) after the neoadjuvant treatment [34]. These results highlight the possible shift of TNBC subtypes during the disease course and underscore that the re-assessment of TNBC subtypes at different timepoints deserves further investigation to assess its potential impact on prognosis and therapeutic tailoring. 

Another limitation for the implementation of these molecular classifications in clinical practice is the access to next-generation sequencing (NGS). To overcome this, an IHC-based approach, easier to apply in a clinical setting, has been developed by analyzing RNA-seq data from TNBC datasets. In this approach, four markers (AR, CD8, FOXC1, and DCLK1) have been selected based on their level of expression in each subtype and their good correlation with protein expression to reproduce the molecular subtyping [35]. This IHC-derived approach to determine different mTNBC subtypes (LAR, IM, BLIS, and MES) was applied in the FUTURE study, a phase Ib/II umbrella trial. In addition, targeted sequencing with a gene panel including *ERBB2*, *BRCA1/2* germline, and mutations involving the *PI3K/AKT* pathway was used to stratify patients into seven treatment arms, including one arm in which a combination of the anti-PD1 ICI camrelizumab and nab-paclitaxel was administered in patients with an IM subtype [36]. In this heavily pre-treated population, IM tumors had an objective response rate (ORR) of 52.6% [36]. The activity of ICI in the IM subtype was also demonstrated in the FUTURE-C-PLUS trial, in which treatment-naive mTNBC patients who were treated with a combination of camrelizumab, famitinib (a multi-targeted tyrosine kinase inhibitor), and nab-paclitaxel obtained an impressive ORR of 81.3% [37].

## 3. Programmed Death-Ligand 1 Protein Expression

In BC, PD-L1 is expressed in 20–50% of primary tumors and only in 15% of metastatic samples. The BC subtypes with the most common PD-L1 expression consist of TNBC and HER2+ BC, which also presented higher TIL levels [38,39]. As expected, PD-L1 is mostly expressed in the immune-related molecular subtypes (BLIA/IM/BL: 33–78%), followed by BLIS (4,7–32%), LAR (0–35%), M (31%), and MES/MSL (0–65%) [32,36,40,41,42,43] (Table 2). However, differences in the thresholds and tests used to define PD-L1 positivity and the use of different TNBC classification systems need to be considered when comparing the different studies (Table 3).

In phase I-II trials in mTNBC, PD-L1 IHC expression was associated with a higher response rate to ICI monotherapy. However, some of these studies only included patients with PD-L1-positive tumors [16,45,46,47,48]. The predictive value of PD-L1 IHC and its association with a better outcome in mTNBC were demonstrated in the phase III IMpassion 130 and KEYNOTE 355 trials evaluating a combination of an anti-PD-(L)1 ICI and chemotherapy as first-line therapy [7,23]. Since 2019, two companion diagnostics for PD-L1 testing have been approved by the Food and Drug Administration (FDA) to select unresectable or metastatic TNBC to be treated with an ICI and chemotherapy [49] (Table 1 and Table S1).

PD-L1 is far from an optimal biomarker. In fact, responses to ICI-based therapy are also observed in patients with PD-L1-negative tumors, and not all patients with PD-L1-positive TNBC benefit from immunotherapy. This inconsistency could be related to tumor heterogeneity (e.g., potential differences in PD-L1 staining which can be observed between primary tumor samples and metastatic lesions, across concurrent metastatic sites, as well as within the same tissue sample), but may also be a result of discrepancies in the PD-L1 assessment methodologies that were used in the different studies [50,51]. Indeed, PD-L1 positivity can be influenced by different aspects of the IHC assay that is being used, including the antibody clone (SP142 vs. 22C3), the staining protocols and platforms (Ventana vs. Dako), the scoring algorithms (immune cells only for SP142 vs. immune and tumor cells for 22C3), and the threshold used for positivity (Combined Positive Score (CPS) ≥10 vs. immune cells (IC) ≥1%). Several reports demonstrated an inconsistent prevalence of PD-L1 positivity across the different clinical trials [52,53,54]. Indeed, each antibody binds to a distinct epitope of PD-L1, leading to specific PD-L1 staining patterns. Indeed, comparable tumor cell staining is obtained with 22C3, 28–8, and SP263, but this is not the case with the SP142 assay, which better identifies immune cells [53,55]. In this regard, deglycosylation of formalin-fixed paraffin-embedded (FFPE) tumor samples can increase cell surface PD-L1 detection, and has been proposed as a method to reduce the proportion of false negative results [56]. Despite these technical considerations, the anatomical site, and the timing of PD-L1 assessment are crucial as well. This is reflected by the higher PD-L1 expression in primary tumors compared to metastatic lesions, or in metastatic lymph nodes compared to liver lesions [8,23]. Furthermore, PD-L1 expression is dynamic, as shown by possible conversions in the PD-L1 status following neoadjuvant chemotherapy [31,57]. 

Interestingly, PD-L1 expression on pre-therapeutic BC biopsies did not predict pCR in the neoadjuvant IMpassion 031 and KEYNOTE 522 trials [1,6]. The benefit obtained by combining chemotherapy with an ICI was in fact consistent across different levels of PD-L1 expression. However, in the GeparNuevo trial, higher pCR rates were observed in patients with PD-L1-positive tumors regardless of the treatment arm, with significant differences when considering the expression of PD-L1 on tumor cells in the durvalumab arm and on immune cells in the placebo arm [24,58] (Table S2). 

The discordance in the predictive value of PD-L1 between the early and metastatic settings may also be related to the immunoediting processes of cancer cells when evolving from a primary tumor to metastatic lesions, and to the progressive development of a TME more conducive to immune evasion. In this respect, the emergence of less immunogenic tumors in combination with a more immunosuppressive TME in metastatic lesions may make the prediction of a response to the ICI in this setting more dependent on biomarkers such as PD-L1 expression [59,60,61].

In conclusion, the indication for PD-L1 testing currently remains limited to mTNBC, and its results should be interpreted with caution given its many flaws as a biomarker. 

Given the technical issues associated with the IHC PD-L1 status, assessing *CD274* gene amplification may potentially be a more robust biomarker. *CD274* encodes for PD-L1, and the presence of gain or amplification of this gene was associated with increased PD-L1 expression (assessed with SP142) in cancer cells but not in immune cells, in line with a cancer cell-intrinsic expression pattern related to this genomic alteration and an adaptative mechanism of immune evasion during cancer evolution [31].

Recently, Lehmann et al. described the distribution of *CD274* amplification across the 4-type molecular TNBC classification, reporting a generally low prevalence. A higher incidence of *CD274* amplifications was observed in the M subtype (11%), followed by the BL2 subtype (5%). However, M tumors displayed increased methylation in the promoter region of the *CD274* gene, which hampered the expression of PD-L1 on the cell surface [9] (Table 2). Interestingly, neoadjuvant chemotherapy could lead to the selection of *CD274* amplification in TNBC, and, thus, to an increase in PD-L1 expression [62]. In the SAFIR02 BREAST IMMUNO phase II trial, PD-L1 CNVs assessed through a CGH array (Affymetrix CytoscanHD or Oncoscan) showed that a gain (3 to 4 copies) or an amplification (≥ 4 copies) of *CD274* was predictive for a benefit from durvalumab in metastatic BC [31] (Table 1).

## 4. Role of Agnostic Biomarkers for ICI Therapy in TNBC

Microsatellite instability (MSI) and TMB are two tissue-agnostic biomarkers approved by the FDA for the use of pembrolizumab in patients with an unresectable or metastatic solid tumor progressing to prior therapy [17,18]. TMB is generally considered as a surrogate for the neoantigen load and a biomarker of T cell activation [20,21,63]. Even if TMB is higher in TNBC compared to other BC subtypes, the overall median TMB in TNBC is low (1.8 mutations per megabase) compared to other tumors [22]. In TNBC, a TMB of >1.5 mut/Mb was significantly associated with a better progression-free interval [9]. Lehmann et al. showed that BL1 and M subtypes harbored more mutations than the BL2 and LAR subtypes [9] (Table 2). Based on the phase II KEYNOTE 158 trial results, a cut-off point of more than 10 mutations/megabase (mut/Mb) of DNA using a targeted sequencing FoundationOneCDx assay (F1CDx) was defined for the use of pembrolizumab monotherapy in advanced solid tumors. [25,64]. Of note, this indication involves 5–10% of patients with TNBC [20,21,22]. The relevance of this biomarker in solid tumors is still controversial, as frequent tumor types such as prostate cancer and microsatellite stable colorectal cancer were underrepresented in the KEYNOTE 158 trial. Furthermore, a recent retrospective analysis showed that when CD8 T cell levels are not correlated to a neoantigen load, a high TMB fails to predict the response to the ICI across different cancer types, including BC [65]. In the multi-basket MyPathway (NCT02091141) study, a higher cut-off of F1CDx TMB of ≥16 mut/Mb demonstrated a larger benefit from atezolizumab monotherapy across a broad spectrum of advanced solid tumors, regardless of the MSI status (ORR = 38.1%). In contrast, limited efficacy was observed in patients with F1CDx TMB ≥10 and <16 mut/Mb (ORR = 2.1%, n = 48) [66]. In BC, an exploratory analysis of the phase III KEYNOTE-119 trial suggested a positive association between TMB-H status and a clinical benefit from pembrolizumab in patients with mTNBC, in line with the phase II TAPUR trial [21,26] (Table 1 and Table S1). In the neoadjuvant GeparNuevo trial, TMB assessed using whole-exome sequencing with a cut-off based on the upper tertile (2.05 mut/Mb) was associated with the pCR in both study arms (with and without ICI), suggesting that TMB is a prognostic rather than a predictive biomarker in this setting [67].

As a consequence of accumulating tumor-specific non-synonymous mutations, the neoantigen load could be an additional biomarker for the response to ICIs, potentially more precise than TMB. As TNBC tumors exhibit a higher mutation rate, they are more likely to harbor neoantigens and higher TIL levels than ER+ BC [26,68,69]. These mutations are foreign to the host genome and can induce adaptive anti-tumor immune responses [70]. In BC, a slightly higher neoantigen load was observed in responders to ICIs with an anti-PD-L1 and anti-CTLA-4 [71]. However, assessing the neoantigen load requires the acquisition of different parameters derived from whole-exome and RNA-seq, including HLA-typing and the prediction of major histocompatibility complex binding, which makes it challenging in routine clinical practice [72].

MSI, characterized by abnormal losses or gains of nucleotides in repetitive microsatellite sequences, is a result of a highly mutagenic tumor phenotype secondary to DNA mismatch repair deficiency [73]. IHC and PCR-based assays are currently used to assess the MSI status [74,75,76,77]. Unlike colorectal or endometrial cancers, high levels of microsatellite instability (MSI-H) or deficient mismatch repair (dMMR) status are extremely rare in TNBC, with only approximately 0.2% of TNBC cases being MSI-H/dMMR [19,77]. In 2017, the FDA approved the use of MSI-H/dMMR as a tissue-agnostic biomarker to select patients eligible for a treatment with pembrolizumab based on a pooled analysis of 149 patients with MSI-H/dMMR cancers enrolled in single-group clinical trials (Table 1) [17,27]. However, only 2 BC patients were included in this study. Up to now, no data about the distribution of MSI status across TNBC molecular subtypes are available.

## 5. Tumor Infiltrating Lymphocytes and Spatial Immune Organization

TILs refer to a variable set of leukocytes, mostly consisting of T cells, with lower proportions of B and natural killer cells [78]. The prevalence of TILs is heterogeneous across different BC subtypes, with TNBC and HER2+ BC exhibiting higher levels of TIL infiltration compared to luminal-like BC subtypes [79]. TILs are commonly scored on hematoxylin and eosin (H&E)-stained tissue slides, and are categorized into stromal (sTILs) and intratumoral (iTILs) TILs [78]. 

According to the Lehmann’s molecular classification, TILs are more abundant in IM tumors (percentage of TILs: 38%), followed by BL2 (23%), MSL (21%), LAR (17%), BL1 (15%), and M (9%) [13] (Table 2). Interestingly, the immune infiltrate composition differs as well across the different molecular subtypes, with adaptive immune cells being enriched in IM, and innate immune cells being more represented in MSL and LAR subtypes [15]. 

An International Immuno-Oncology Biomarker Working Group on Breast Cancer developed guidelines to standardize TIL scoring in BC in order to increase the reproducibility of TILs’ assessment and to facilitate their integration and interpretation in clinical trials [80,81,82]. However, in the era of artificial intelligence (AI), novel tools such as automated TIL scoring systems using machine and deep learning approaches are in development, aiming to overcome the inherent inter-operator variability of visual TIL estimation [83,84,85]. Importantly, the integration of this biomarker in the traditional TNM American Joint Committee on Cancer (AJCC) Staging System for eTNBC is under discussion [86]. In parallel, an online tool is actually accessible to determine the prognosis based on variables such as TILs, nodal status, age, and tumor size [87]. A growing body of evidence has confirmed the prognostic and predictive values of TILs in TNBC patients undergoing standard treatments, which formed the rationale to also interrogate their clinical utility in the context of ICI therapy.

In the metastatic setting, higher TIL levels have been associated with a better ORR to single-agent ICI and to a better overall survival (OS) in TNBC patients [28,29,44]. In the phase III IMpassion130 trial, the combination of atezolizumab and nab-paclitaxel proved to be associated with a longer PFS in patients whose tumors harbored sTILs ≥10% (HR: 0.64, 95% CI = 0.5–0.84) (Table 1 and Table S1). This difference was even more pronounced when TIL levels were combined with PD-L1 positivity (HR: 0.54, 95% CI = 0.39–0.75) [23]. 

Dynamic monitoring between baseline and on-treatment biopsies in the TONIC trial also showed higher TILs and CD8+ lymphocytes in responders compared to non-responders among advanced TNBC patients treated with nivolumab monotherapy after an immune induction phase [88] (Table S1). 

In early BC, higher sTIL levels were associated with a better response to the ICI in the NeoTRIPaPDL1, GeparNuevo, and KEYNOTE 173 trials [24,30,57] (Table S2). In addition, baseline iTIL levels were found to be associated with a higher pCR rate following treatment with a combination of chemotherapy and atezolizumab in the NeoTRIPaPDL1 trial. Importantly, the median increase in sTILs and iTILs between pre-treatment and on-treatment samples was higher in patients who achieved a pCR in the KEYNOTE 173 and GeparNuevo trials, illustrating the potential value of TIL dynamics in predicting ICI efficacy [30,57]. However, given the poor reproducibility of iTILs estimations, we must interpret these results with caution [82]. Recently, the neoadjuvant phase II BELLINI trial was the first study to use TIL levels to select highly immunogenic early-stage TNBC for treatment with nivolumab ± low-dose ipilimumab, and to identify a subgroup of patients who would benefit from a therapeutic de-escalation. Both arms of this study met their biomarker-based primary endpoint, with an increase in CD8+ T cells and/or IFN-gamma expression in 53% of patients in the nivolumab cohort and of 60% in the nivolumab/ipilimumab cohort. In this trial, responders had baseline sTILs levels of ≥40% with a concentration of CD8+ T cells in the proximity of tumor cells (*p* = 0.0014) [89].

Beyond the broad assessment of TILs in TNBC, specific immune cell compositions were shown to influence the response to the ICI as well. In this respect, CD8+ T cells seem to be the most important cell subset. In-depth analyses of TIL compositions showed an association between CD8+ T cells and T regulatory cells with a better outcome under chemo-immunotherapy across different trials, including a phase Ia study (NCT01375842) and the TONIC, I-SPY2, and KEYNOTE 086 trials [44,88,90,91]. Interestingly, single-cell data suggested a potential association between tertiary lymphoid structures, which are ectopic lymph node-like structures characterized by lymphoid aggregation and colocalization with CXCL13 + CD8+ T cells, CD4+ T cells, and CXCR5+ B cells, and the response to the ICI [92].

Recently, data supporting the importance of the immune cell contextures within the tumor microenvironment led investigators to look into the potential prognostic role of the spatial localization of TILs in TNBC [23,93]. At least three spatial immunophenotypes have been identified: a fully inflamed (FI) phenotype (characterized by intratumoral localization of TILs), a stroma-restricted (SR) phenotype (absence of iTILs, but presence of TILs in the stroma), and a margin-restricted (MR) phenotype (presence of TILs at tumor margins). The latter of these phenotypes could encompass immune desert (ID) tumors (low abundance of TILs). Regarding the TNBC molecular classifications, a higher proportion of FI tumors was found in the IM subtype (46–76%) [15,43]. In contrast, the SR phenotype was mainly observed in BL/BL1 subtypes (62.5–78%), while it was less frequent in the BL2 subtype (0–27%). The MR phenotype was instead more prevalent in the LAR and M subtypes (33–43% and 41–93%, respectively). Finally, the M subtype likely had the lowest immunogenic potential, with lower levels of TIL infiltration and a higher prevalence of the MR or ID phenotype compared to the other subtypes [9,13,15,43] (Table 2).

The impact of different spatial immunophenotypes on the therapeutic response is increasingly being explored in clinical trials evaluating ICIs in TNBC. In IMpassion130, FI- and immune-excluded tumors, defined by a significant CD8+ T cell infiltration in the tumor or in the stroma compartment, respectively, were associated with better outcomes in the atezolizumab arm compared to ID tumors. In addition, a particularly prolonged OS was observed in FI tumors which were treated with atezolizumab [29]. 

Moreover, a high degree of spatial connectivity between epithelial and specific TME cell phenotypes (e.g., CD8 + PD1 + TEX T cells, CD8 + GZMB+ T cells, CD20+ B cells) proved to be associated with a higher pCR rate when adding atezolizumab to chemotherapy in the neoadjuvant NeoTRIPaPDL1 trial, independent of PD-L1 expression and the number of sTILs [94]. Of note, dynamic monitoring in the TONIC trial showed an increase in the proportion of inflamed phenotypes after induction treatment with cisplatin and doxorubicin. This illustrates that the TME can change under chemotherapeutic pressure, a feature that can potentially be exploited to enhance ICI activity [95].

## 6. Gene Expression Signatures

The first generation of molecular analyses in BC based on gene expression profiling identified several gene signatures driven by proliferating genes, and improved the prognosis prediction [96]. Originally, immune-related genes were considered as confounding variables within microarray-based gene expression analyses. However, in recent years, the immune response evaluated by the expression of immune genes was demonstrated to be a major molecular process associated with prognosis, especially in HER2+ and TNBC subgroups, and was included in the BC taxonomy [97]. Immune gene signatures reflect the relative abundance of tumor-infiltrating immune cells and define subpopulations of immune cells as well as several immunological features that exhibit a significant correlation with patient outcome and therapeutic response [98,99]. As the RNA-seq of tumor samples usually encompasses both tumor cells and cells from the microenvironment, researchers have developed expression profile-based tools for the relative or absolute estimation of the abundance of microenvironment cells in tumor tissues (e.g., Cibersort, MCP-counter) [100,101]. These tools are able to identify different immune subsets in tumor-derived samples using specific immune gene expression signatures and/or marker genes.

In mTNBC, B and T cells’ gene expression signatures were significantly associated with better outcomes in patients treated with atezolizumab monotherapy [28]. In addition, the 18-gene T cell-inflamed gene expression profile (GEP) and the 37-gene tissue-resident memory (TRM) T cell signature proved to be associated with the response to pembrolizumab [102]. More recently, data suggested that the predictive value of the TRM signature in ICI-treated patients may be explained by the exhausted phenotype of TRM cells, which could be re-activated by the addition of ICI ex vivo [103]. In eTNBC, higher levels of dendritic cell and STAT1_sig/chemokine12 gene signatures have been linked to the response to pembrolizumab [104]. In the GeparNuevo phase II trial, the GeparSixto immune gene expression signature (GSIS), TMB, and an interferon signature were independently predictive for pCR following treatment with a durvalumab-chemotherapy combination in the neoadjuvant setting [58,67]. Of note, GSIS is composed of 12 immune genes that differentiate “immune-hot” from “immune-cold” tumors, and include both immune-activating genes (*CCL5*, *CXCL9*, *CXCL13*, *CD80*, *CD21*, *CD8A*, *IGKC*) and immunosuppressive genes (*PDCD1*, coding for PD-1, *CD274*, coding for PD-L1, *CTLA4*, *FOXP3*, and *IDO1*). The prevalence of specific immune signatures has rarely been assessed in the function of the TNBC molecular classification. Indeed, only GSIS was evaluated across different molecular subtypes in a phase II trial assessing the activity of neoadjuvant carboplatin and nab-paclitaxel in eTNBC [44]. Immune-hot tumors were identified in 100% of IM, 45% of BL1, and 40% of BL2 subtypes. In line with previous results, only 1 LAR (16%), 1 MSL (25%), and no M tumors were characterized as being immune-hot [44] (Table 2). 

More recently, the NeoTRIPaPDL1 trial highlighted the predictive value of a 27-gene immuno-oncology (IO) score, and of a B cell memory signature for a response to atezolizumab combined with chemotherapy [32]. Interestingly, the dynamics of the IO score computed on biopsies collected early during treatment were linked to the likelihood for a pCR independently of baseline biomarkers, and may be an early surrogate for treatment benefit in patients receiving immunotherapy [105]. 

However, these signatures are largely redundant, and do not account for the spatial distribution of immune cells, nor do they allow the analysis of detailed features that can be captured using spatial transcriptomics or single-cell analyses [16,17,18,19,20,21,22,23,24,25,26,27,28,29,30,31,32,33,34,35,36,37,38,39,40,41,42,43,44,45,46,47,48,49,50,51,52,53,54,55,56,57,58,59,60,61,62,63,64,65,66,67,68,69,70,71,72,73,74,75,76,77,78,79,80,81,82,83,84,85,86,87,88,89,90,91,92,93,94,95,96,97,98,99,100,101,102,103,104,105,106,107,108]. Of note, the implementation of gene expression signatures using RNA-seq is challenging and comes with additional costs. Recently, alternatives to RNA-seq, such as a quantitative reverse transcription-polymerase chain reaction test for IO score assessment, were confirmed to be predictive of the atezolizumab benefit over chemotherapy alone in the NeoTRIPaPDL1 trial [105]. 

In addition to immune signatures, several canonical cancer pathways were recently shown to be associated with outcome following ICI therapy. I-SPY 2 investigators tested 9 gene expression signatures reflecting different aspects of DNA damage and repair. Of these biomarkers, a MammaPrint High2 status and DNA damage sensing pathway including the *ATM*, *ATR*, *CHEK1*, and *CHEK2* genes proved to be associated with a response to pembrolizumab [109]. Of interest, the aforementioned amplification of *CD274* can be associated with *JAK2* amplification in around 10% of TNBC cases after neoadjuvant treatment, making it a potential predictor for a response to ICI therapy [110]. Recently, data from the TONIC trial showed that a short-term treatment with doxorubicin and cisplatin can reprogram the TME by up-regulating the *JAK-STAT* and TNF-α signaling, resulting in a higher sensitivity to nivolumab in mTNBC [88]. Finally, in the IMpassion130 trial, different hallmarks including processes involved in DNA repair and proliferation (e.g., “DNA repair”, “G2/M Checkpoint”, or “Mitotic Spindle”) were associated with a better PFS in the atezolizumab arm [29]. 

However, the predictive role of these different pathways needs extensive prospective validation in BC treated with ICIs.

## 7. Discussion and Conclusions

Over the past decades, advances in cancer immunotherapy have significantly improved the prognosis of many patients with a variety of malignancies. Meanwhile, efforts are underway to better understand the mechanisms associated with treatment response and resistance. These insights will allow physicians to better select the most suitable treatment strategy for each patient. As summarized in this review, TNBC heterogeneity should be considered in the development of predictive biomarkers, and multiple challenges are to be faced for the implementation of immune “precision therapy” in patients with TNBC. 

Despite its biological and clinical heterogeneity, only two biomarkers (PD-L1 IHC staining for immunotherapy and germline *BRCA1/2* mutations for PARP inhibitors) are currently available to tailor therapy in TNBC. The TNBC molecular subtypes established in the past decade through extensive genomic and transcriptomic analyses are an important achievement and allow us to better categorize TNBC and identify targetable pathways [10,11,12,13,14,15]. However, this TNBC classification is still largely theoretical and is not yet used in clinical practice. Indeed, molecular subtyping requires RNA-sequencing of the tumor sample which is costly and not widely accessible. However, some recent studies are considering molecular TNBC subtypes either as an inclusion criterion or as a biomarker for subgroup analyses [29,36,37,40]. Other limitations to implement TNBC subtypes in clinical practice are the absence of consensus between the different classification systems and the fact that none of them has been validated in the metastatic setting. Of note, comparisons of the genomic and transcriptional characteristics of primary tumors and corresponding relapses as well as their molecular subtypes between early and metastatic TNBC settings are warranted for a better understanding of this multi-faceted disease. The AURORA study performing multi-omics profiling with paired primary tumors and early-course metastases in BC may address this issue [111]. In parallel, other studies are exploring new approaches using IHC surrogates for TNBC molecular classification as used in the FUTURE trial, or imaging-based deep learning models [35,36,112,113]. Further studies investigating the ability of these surrogates to determine each subtype are needed. 

Despite all these efforts, the TNBC heterogeneity is still not completely deciphered, and this heterogeneity has yet to be considered in the development of predictive or prognostic biomarkers. This review clearly illustrates the variable potential of biomarker candidates between different TNBC subtypes, which should be addressed as distinct diseases, particularly with regards to treatment with the ICI [9,13,15,29,32,40,41,42,43,44]. In addition to the molecular heterogeneity, the development of biomarkers predictive for a response to the ICI in TNBC is challenging and limited by several technical aspects. This includes a lack of standardization between studies investigating potential biomarkers, a fact that is amply illustrated by PD-L1 IHC expression [54,55]. As a result, comparing the results from different studies is unreliable, and determination of the clinical significance of the biomarker is challenging. 

**Figure 3:**
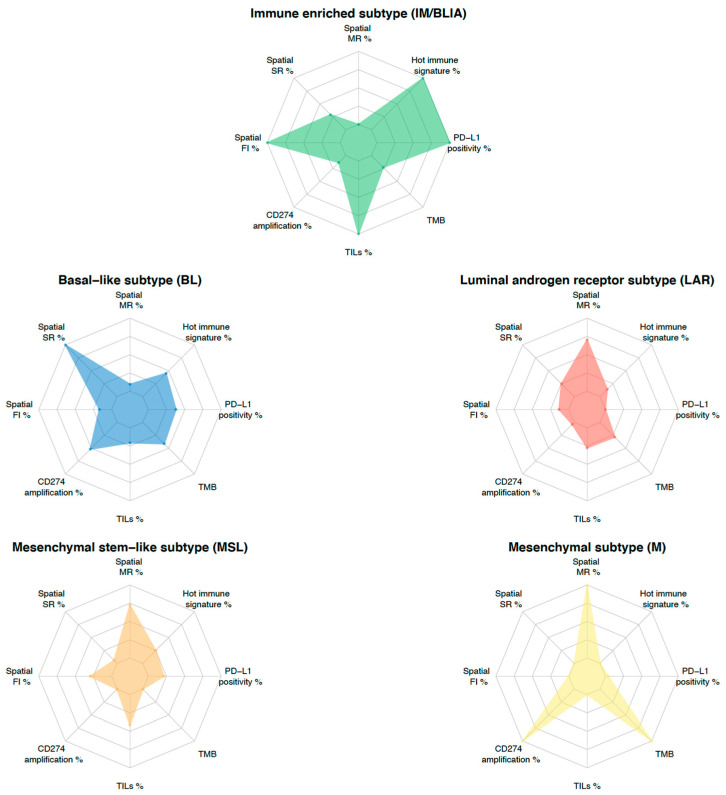
TNBC molecular subtyping immunogram. The radar plot depicts eight parameters that characterize biomarkers potentially predicting a response to ICI-based therapy. Levels of each biomarker are rescaled between minimum (internal ring) and maximum value (external ring) for each parameter across subtypes. The contribution of each biomarker to TNBC molecular subtype is delineated by the connected colored line. Each subtype is represented by a specific color. Abbreviations. FI: fully inflamed, MR: margin-restricted (including immune desert), PD-L1: programmed death-ligand 1, SR: stroma-restricted, TILs: tumor infiltrating lymphocytes, TMB: tumor mutational burden (mut/Mb).

To overcome the challenges posed by tumor heterogeneity, an integrative approach combining multiple predictive biomarkers is emerging as a new strategy for the development of biomarkers. For instance, the combination of PD-L1 expression and TILs with or without an 18-gene T cell-inflamed GEP, TMB, CD8 IHC, and a glycolysis signature emphasized the additional predictive value to the ICI in the KEYNOTE 086, GeparSixto, and IMpassion130 trials [16,92,114,115]. This integrative approach could be transposed to TNBC molecular classification. To this end, each molecular feature could be evaluated through an individualized TNBC immunogram, as proposed by Blank et al. [116]. In this regard, as shown in Figure 3, different TNBC subtypes inherently present different features. This subtype-guided immunogram could help to deeply dissect tumor heterogeneity by highlighting the different contributions of the previously described predictive biomarkers and to better tailor future immune “precision therapy” in TNBC. Indeed, specific biomarkers integrated with molecular subtypes may provide a more comprehensive evaluation. It is now obvious that to improve the use of immunotherapy in TNBC, we have to develop clinical trials for targeted populations driven by biomarkers. 

In addition, retrospective translational research analyses evaluating multiple biomarkers in immunotherapy trials will help to advance the development and validation of these biomarkers. However, this requires easier access to clinical trial data across the whole research community. On the other hand, new prospective studies will be needed to validate each biomarker or their combination in predictive models gathering patient- and tumor-intrinsic characteristics along with dynamic immune parameters. 

A next step in this biomarker development will come from data provided by novel technologies that are able to depict the TME (e.g., spatial transcriptomics/proteomics and single-cell sequencing) at an unprecedented level [95,106,108,117,118] and by data supporting novel biomarkers in other cancer types [119,120,121]. For instance, the characterization of both gut and breast microbiomes could lead to further advancements in the prediction of the response to the ICI in TNBC [122,123]. In BC, distinct microbial signatures are associated with BC subtypes [124]. Moreover, breast microbes may also modulate the tumor microenvironment and the immune activation in BC patients, providing opportunities to target microbes to improve outcomes and the prediction of treatment response [123]. Interestingly, diet could influence the microbes in the gut, and a recent study demonstrated that patients reporting sufficient fiber intake (>20 g/day) have better outcomes on the ICI in melanoma patients [124]. Furthermore, liquid biopsies, having the advantage of being conservative procedures, could also be used to predict and actively monitor the response to immunotherapy. Indeed, in TNBC, higher circulating T cell receptor clonality/diversity, baseline circulating tumor DNA levels, and kinetics were associated with a clinical benefit to the ICI [88,125] (Figure 1). The full potential of these new tools needs to be further explored in prospective randomized trials. 

To conclude, as highlighted by the data reviewed in this manuscript, it is clear that TNBC heterogeneity should be considered when evaluating new biomarkers in this setting. In the future, an approach considering TNBC as a multi-faceted disease will help in the development of more tailored therapies in line with patient-centered care. 

## Figures and Tables

**Table 1:** Clinically approved and evolving predictive biomarkers for a response to ICIs.

Biomarker	Assay	Platform	Location	Prevalence	Description	Cutoff	Clinical Trial	ICI	BC Subtype	FDA Approval
PD-L1	Dako 22C3	Agilent	TC + IC	20–50% of all BC	CPS score = (PD-L1 + ) IC + (PD-L1 + ) TC × 100/TC	CPS score + ≥ 10%	Phase III KEYNOTE 355 [7]	Pembro-lizumab	mTNBC	mTNBC
	Ventana SP142	Roche	IC		IC score = % tumor area with IC labeling with PD-L1 at any intensity	IC score + ≥ 1%	Phase III IMpassion 130 [23]	Atezoli-zumab	mTNBC	mTNBC
	Ventana SP263		TC + IC		Score = % TC + % IC labeling with PD-L1 at any intensity	PD-L1 + ≥ 1% in TC and/or IC	Phase II GeparNUEVO [24]	Durva-lumab	eTNBC	No
TMB	F1CDx	Founda-tion Medicine	TC	5–10% of TNBC	Total number of synonymous or non-synonymous mutations for 324 cancer-related genes	High TMB ≥ 10 mut/Mb	Phase II KEYNOTE 158 [25]	Pembro-lizumab	mBC	All BC
							Phase III KEYNOTE 119 [21]	Pembro-lizumab	mTNBC	No
						High TMB ≥ 9 mut/MB	Phase II TAPUR [26]	Pembro-lizumab	mBC	No
MSI/dMMR	IHC targe-ting MLH1, MSH2, MSH6, and PMS2	Ventana; Promega	TC	0.04–1.8% of TNBC	Targeting five monomorphic mononucleotide repeat markers (BAT-25, BAT-26, MONO-27, NR-21, and NR-24) for MSI typing and two highly polymorphic pentanucleotide repeat markers (Penta C and Penta D) for sample identification	High MSI: at least 2 unstable markers out of 5 (≥ 40% of MS markers)	Pool analysis of 5 trials: KEYNOTE 016, 164, 012, 028, 158 [27]	Pembro-lizumab	mBC	All BC
	PCR with MSI Analysis System									
Stromal TILs	H&E staining	/	IC	Total TILs: up to 75% of all BC; LPBC: up to 20% BC	sTILs = % of intratumoral stromal compartment occupied by TILs	sTILs ≥ 5%	Phase III KEYNOTE 119 [28]	Pembro-lizumab	mTNBC	No
							Phase II KEYNOTE 086 [29]			
						sTILs ≥ 10%	Phase III IMpassion 130 [23]	Atezoli-zumab	mTNBC	No
							Phase Ib PCD4989 g [16]			
						sTILs > 40%	Phase Ib KEYNOTE 173 [30]	Pembro-lizumab	eTNBC	No
						On treatment sTILs ≥ 40%	Phase III NeoTRIPaPDL1 [31]	Atezoli-zumab	eTNBC	No
						On treatment sTILs ≥ 65%	Phase Ib KEYNOTE 173 [30]	Pembro-lizumab	eTNBC	No
Intra-tumoral TILs	H&E staining	/	IC	Total TILs: up to 75% of all BC; LPBC: up to 20% BC	iTILs = lymphocytes in tumor nests having cell-to-cell contact with no intervening stroma and directly interacting with carcinoma cells	Dynamic change of iTILs between baseline and after the window-phase	Phase II Gepar-NUEVO [24]	Durva-lumab	eTNBC	No
						Baseline high-/intermedia-te iTILs	Phase III NeoTRIPa-PDL1 [31]	Atezoli-zumab	eTNBC	No
CD274 gene amplifi-cation	CGH array	Affyme-trix CytoscanHD/Onco-scan	TC	Up to 30–42% of TNBC	PD-L1 gene copy = loss, neutral, or copy gain/amplification	Gain: 3 or 4 copies; amplifica-tion: ≥ 5 copies	Phase II SAFIR02-IMMUNO trial [32]	Durva-lumab	mTNBC	No

Abbreviations. BC: breast cancer, CGH: comparative genomic hybridization, CPS: combined positive score, eTNBC: early triple negative breast cancer, F1CDx: FoundationOne CDx, H&E: hematoxylin and eosin, IC: immune cells, ICI: immune checkpoint inhibitor, IHC: immunohistochemistry, iTILs: intratumoral tumor infiltrating lymphocytes, LPBC: lymphocyte predominant breast cancer, MS: microsatellite, MSI/dMMR: microsatellite instability/mismatch repair deficiency, mBC: metastatic breast cancer, mTNBC: metastatic triple negative breast cancer, PCR: polymerase chain reaction, PD-L1: programmed death-ligand 1, sTILs: stromal tumor infiltrating lymphocytes, TILs: tumor infiltrating lymphocytes, TC: tumoral cells, TMB: tumor mutational burden, TNBC: triple negative breast cancer.

**Table 2:** Distribution of biomarkers across different TNBC molecular subtypes.

Predictive Biomarker	Study	Total Number (N)	BL-Related					LAR	Mesenchymal- Related	
			BL	BLIA-IM	BLIS	BL1	BL2	LAR	M	MSL-MES
PD-L1 positivity										
Percentage of PD-L1 positivity	Sood et al. [41]									
		N = 119	18/28 (64%)	-	-	-	-	8/34 (24%)	-	15/57 (26%)
	Alves et al. [42]									
		N = 57	-	11/33 (33%)	1/21 (4.7%)	-	-	0/2 (0%)	-	0/1 (0%)
	Phase III IMpassion 130 [29]									
		N = 836	-	167/226 (74%)	112/351 (32%)	-	-	67/217 (31%)	-	12/42 (28%)
	Phase III NeoTRIPaPDL1 [32]									
		N = 227	-	-	-	64/82 (78%)	11/19 (58%)	12/34 (35%)	17/56 (31%)	23/36 (65%)
	Phase II FUTURE [36]									
		N = 19	-	13/19 (67%)	-	-	-	-	-	-
	Phase II Pembrolizumab + Enobosarm [40]									
		N = 16	-	-	-	-	-	2/16 (12.5%)	-	-
Tumor mutational burden										
TMB (mut/Mb)	Lehmann et al. [9]									
		N = 183	-	-	-	N = 64 (2.1 mut/Mb)	N = 37 (1.2 mut/Mb)	N = 28 (1.8 mut/Mb)	N = 54 (2.3 mut/Mb)	-
Tumor infiltrating lymphocytes rate										
Percentage of TILs	Lehmann et al. [13]									
		N = 167	-	N = 36 (TILs: 38%)	-	N = 34 (TILs: 15%)	N = 17 (TILs: 23%)	N = 18 (TILs: 17%)	N = 40 (TILs: 9%)	N = 22 (TILs: 21%)
Distribution of spatial immunophenotype										
Percentage of each immunophenotype	Bareche et al. [15]									
	FI (%)	N = 697	15/162 (9%)	138/181 (76%)	-	-	-	11/124 (9%)	0/141 (0%)	19/89 (21%)
	SR (%)		126/162 (78%)	41/181 (22%)	-	-	-	26/124 (21%)	9/141 (6%)	4/89 (4.4%)
	MR (%)		21/162 (13%)	2/181 (1%)	-	-	-	87/124 (70%)	132/141 (93%)	66/89 (74%)
Percentage of each immunophenotype	Gruosso et al. [43]									
	FI (%)	N = 31	-	5/11 (46%)	-	1/8 (12.5%)	1/2 (50%)	0/3 (0%)	0/6 (0%)	0/1 (0%)
	SR (%)		-	4/11 (36%)	-	5/8 (62.5%)	0/2 (0%)	1/3 (33%)	0/6 (0%)	1/1 (100%)
	MR (%)		-	2/11 (18%)	-	2/8 (25%)	1/2 (50%)	1/3 (33%)	4/6 (67%)	0/1 (0%)
	ID (%)		-	0/11 (0%)	-	0/8 (0%)	0/2 (0%)	1/3 (33%)	2/6 (33%)	0/1 (0%)
Percentage of each immunophenotype	Lehmann et al. [9]									
	FI (%)	N = 183	-	-	-	14/64 (22%)	9/37 (24%)	5/28 (18%)	1/54 (2%)	-
	SR (%)		-	-	-	28/64 (44%)	10/37 (27%)	8/28 (29%)	4/54 (7%)	-
	MR (%)		-	-	-	17/64 (27%)	10/37 (27%)	12/28 (43%)	22/54 (41%)	-
	ID (%)		-	-	-	5/64 (8%)	8/37 (22%)	3/28 (11%)	27/54 (50%)	-
Immune signature prevalence										
Percentage of immune-hot GSIS signature	Phase II Carbo + Nabpaclitaxel [44]									
		N = 58	-	16/16 (100%)	-	5/11 (45%)	2/5 (40%)	1/7 (14%)	0/15 (0%)	1/4 (25%)
CD274 amplification rate										
Percentage of CD274 amplification	Lehmann et al. [9]									
		N = 183	-	-	-	6/64 (9%)	2/37 (5%)	2/28 (7%)	6/54 (11%)	-

Abbreviations. BL: basal-like, BLIA: basal-like immune-activated, BLIS: basal-like immune-suppressed, GSIS: GeparSixto immune gene expression signature, IM: immunomodulatory, LAR: luminal androgen receptor, M: mesenchymal, MES: mesenchymal, MSL: mesenchymal stem-like, PD-L1: programmed death-ligand 1, TILs: tumor infiltrating lymphocytes, TMB: tumor mutational burden.

**Table 3:** Factors contributing to the variability of PD-L1 testing and interpretation across TNBC molecular subtypes.

	PD-L1 Clone	PD-L1 Scoring System	TNBC Molecular Classification System		TNBC Staging
			Method	Molecular Subtype	
Sood et al. [41]	22C3	IC	IHC (AR, CK5/6, CK14, claudin 3 and 7, vimentin, e-cadherin, EGFR)	BL, MES, LAR, mixed, unclassifiable	eTNBC
Alves et al. [42]	SP142	TC	IHC (AR, CK5, claudin, *p*-cadherin, EGFR), H&E (TILs)	Burstein’s classification	eTNBC
Phase III IMpassion 130 [29]	SP142	IC	RNA sequencing	Burstein’s classification	mTNBC
Phase III NeoTRIPaPDL1 [32]	SP142	IC	RNA sequencing	TNBCtypes by 101-gene algorithm	eTNBC
Phase II FUTURE [36]	SP142	IC, TC	IHC (AR, CD8, FOXC1)	LAR, IM, BLIS, MES	mTNBC
Phase II Pembrolizumab + Enobosarm [40]	22C3	IC	IHC (AR)	LAR	mTNBC

Abbreviations. AR: androgen receptor, BL: basal-like, BLIS: basal-like immune-suppressed, CK: cytokeratin, EGFR: epidermal growth factor receptor, FOXC1: forkhead Box C1, H&E: hematoxylin and eosin, IC: immune cells, IHC: immunohistochemistry, IM: immunomodulatory, LAR: luminal androgen receptor, MES: mesenchymal, TC: tumor cells, TILs: tumor infiltrating lymphocytes, eTNBC: early triple negative breast cancer, mTNBC: metastatic triple negative breast cancer.

## Data Availability

Not applicable.

## Supplementary Materials

The following supporting information can be downloaded at: https://www.mdpi.com/article/10.3390/jcm12030953/s1, Table S1: Description of major clinical trials assessing immune checkpoint inhibitors in metastatic triple negative breast cancer with biomarker assessment; Table S2: Description of major clinical trials assessing immune checkpoint inhibitors as neoadjuvant treatment in triple negative breast cancer with biomarker assessment.

## Appendix A

Bioinformatic analyses were performed on four publicly available datasets composed of 294 TNBC samples from the Molecular Taxonomy of Breast Cancer International Consortium (METABRIC) [25], 170 TNBC samples from The Cancer Genome Atlas Consortium (TCGA) [26], 360 TNBC samples from patients with Asian ancestry retrieved from Jiang and colleagues [12], and 520 TNBC samples from the Gene Expression Omnibus platform (GSE31519) [27].

- Data acquisition

The breast cancer dataset disclosed by the METABRIC study was downloaded from https://www.cbioportal.org, accessed on 18 December 2022. It contained a normalized RNA microarray profiling of 1992 fresh-frozen breast cancer samples performed on the Illumina HT-12 v3 arrays. We considered as TNBC the BC that had negative results for both HER2 and ER.

The breast cancer dataset disclosed by the TCGA study was hosted by the Broad Institute and deposited in the FIREHOSE Broad GDAC at https://gdac.broadinstitute.org, Access date: 24th September 2020. It contained a raw count from the mRNA-sequencing of 1093 breast cancer samples performed on the Illumina HiSeq 2000. We considered as TNBC the primary BC samples that had negative results for both HER2 and ER.

For the breast cancer dataset retrieved from Jiang and colleagues, RNA-Seq processed data were fetched from the National Omics Data Encyclopedia (NODE) (http://www.biosino.org/node, accessed on 18 December 2022) under accession number (OEP000155).

Gene expression data from TNBC samples disclosed by Rody and colleagues were retrieved from the Gene Expression Omnibus platform (GSE31519) derived from Affymetrix U133A arrays.

- Molecular subtyping

a. Lehmann’s TNBC type-6

This classification is composed of six stable subtypes, namely: two basal-like subtypes (BL1 and BL2), an immunomodulatory subtype (IM), a luminal androgen receptor subtype (LAR), a mesenchymal subtype (M), and a mesenchymal stem-like subtype (MSL). Lehmann’s molecular subtypes were assigned using a reimplementation of the published method, based on the published list of genes positively and negatively associated with each subtype [10]. Briefly, each gene was first normalized to a mean of 0 and a standard deviation of 1. Positive and negative signatures were calculated for each subtype as the mean of the genes positively or negatively associated with that subtype. A subtype score was obtained using the difference between those positive and negative signatures. Each TNBC sample was assigned to the TNBC molecular subtype with the highest score. The main difference with the published method is the lack of the unstable subtype, as each sample was associated with a subtype.

b. Bareche’s molecular subtypes

As previously described by Bareche et al. [14], samples classified from Lehmann’s TNBC type-6 as BL2 and UNS were reclassified using the second highest score. Within this review, the basal-like 1 (BL1) subtype will be referred to as the basal-like (BL) subtype.

c. Burstein’s molecular subtypes

Burstein’s molecular classification is composed of four stable subtypes, namely: a basal-like immune-suppressed subtype (BLIS), a basal-like immune-activated subtype (BLIA), a luminal androgen receptor subtype (LAR), and a mesenchymal subtype (MES). Burstein’s molecular subtypes were assigned in our four datasets by applying the method from PMC6349443. In brief, we rescaled the values from each gene by replacing it by its quantile, leading to values between 0 and 1. We then calculated the Spearman correlations between those rescaled values and the published prototypes, and assigned each sample to the class with the highest correlation.
