# Supplementary material for: Predictive Biomarkers for Response to Immunotherapy in Triple Negative Breast Cancer: Promises and Challenges
Source: J Clin Med. 2023 Jan 26;12(3):953. doi: 10.3390/jcm12030953 (PMC9917763; doi:10.3390/jcm12030953)
Supplement: Supplementary file 1 [file jcm-12-00953-s001.zip › jcm-2140039-SI.pdf]

Supplementary Table S1: Description of major clinical trials assessing ICIs in metastatic TNBC with biomarkers assessment.

	PCD4989g	TONIC	Keynote 086	Keynote 119	Keynote 355	IMpassion 130	IMpassion 131
**Study Design**	Phase Ib	Phase II	Phase II	Phase III	Phase III	Phase III	Phase III
	NCT01375842	NCT02499367	NCT02447003	NCT02555657	NCT02819518	NCT02425891	NCT03125902
	Atezolizumab monotherapy	Randomization into 5 arms:<br>-Nivolumab without induction<br>-Radiotherapy + nivolumab<br>-Cyclophosphamide + nivolumab<br>-Cisplatine + nivolumab<br>-Doxorubicine + nivolumab	Pembrolizumab monotherapy	Pembrolizumab monotherapy vs chemotherapy of choice of investigator (capecitabine, eribulin, gemcitabine, or vinorelbine)	Pembrolizumab and chemotherapy of choice of investigator (nabpaclitaxel, paclitaxel, or gemcitabine plus carboplatin) vs placebo and chemotherapy)	Atezolizumab and nab-paclitaxel vs nab-paclitaxel and placebo	Atezolizumab and paclitaxel vs paclitaxel and placebo
	N = 116	N = 70<br>Maximum of 3 previous line of systemic treatment	N = 254 (all patients)<br>Cohort A (previously treated patients), N = 170<br>Cohort B (previously untreated patients and PD-L1 positive tumor), N = 84	N = 622<br>Previously treated patients with an anthracycline or a taxane in the (neo)adjuvant or metastatic setting	N = 847<br>Previously treated patients with (neo)adjuvant setting allowed	N = 902	N = 651
**Prior line(s) of therapy in metastatic setting**	No prior therapy:<br>N = 21 (18%)<br>1 prior therapy:<br>N = 28 (24%)<br>≥ 2 prior therapies:<br>N = 67 (58%)	No prior therapy:<br>N = 17 (24%)<br>1 prior therapy:<br>N = 34 (49%)<br>≥ 2 prior therapies:<br>N = 19 (27%)	1 prior therapy:<br>N = 53 (31.2%)<br>2 prior therapies:<br>N = 43 (25.3%)<br>≥3 prior therapies:<br>N = 74 (43.5%)	1 prior therapy:<br>N = 374 (60%)<br>2 prior therapies:<br>N = 247 (40%)	No previous chemotherapy or targeted therapy for metastatic TNBC	No previous chemotherapy or targeted therapy for metastatic TNBC	No previous chemotherapy or targeted therapy for metastatic TNBC
**Biopsy requirement for**	Newly collected biopsy or archival tissue	Newly collected biopsy at baseline, post-induction and	Newly collected biopsy (mostly from metastases):<br>N = 146 (75%)	Newly collected biopsy or archival tissue allowed	Newly collected biopsy or archival tissue	Newly collected biopsy or archival tissue:	Newly collected biopsy or archival tissue:

**biomarker assessment**		during immunotherapy	Archival tissue (mostly from primary breast tumors):<br>N = 47 (25%)			Primary breast tumor samples: N = 563<br>Metastatic tumor samples: N = 337	Primary breast tumor samples: N = 336<br>Metastatic tumor samples: N = 315
**PD-L1 testing and scoring**	IHC Ventana SP142<br>PD-L1 scored on ICs:<br>IC0 <1%<br>IC1 ≥1% and <5%<br>IC2 ≥ 5% and <10%<br>IC3 ≥ 10%	IHC 22C3	IHC 22C3<br>Positive if CPS ≥ 1	IHC 22C3<br>Positive if CPS ≥1	IHC 22C3<br>Positive if CPS ≥ 1 and CPS ≥ 10	IHC Ventana SP142<br>Positive if PD-L1 IC ≥ 1%	IHC Ventana SP142<br>Positive if PD-L1 IC ≥ 1%
**PD-L1 population**	IC0: N = 21 (18%)<br>IC1: N = 17 (15%)<br>IC2: N = 49 (42%)<br>IC3: N = 25 (22%)	PD-L1 IC ≥ 1%:<br>N = 60 (86%)<br>PD-L1 IC ≥ 5%:<br>N = 47 (67%)<br>PD-L1 TC ≥ 1%:<br>N = 44 (63%)<br>PD-L1 TC ≥ 5 %:<br>N = 23 (33%)	Cohort A<br>CPS ≥ 1:<br>N = 105 (62%)<br>Cohort B<br>CPS ≥ 1: N = 84 (100%)	CPS < 1: N = 217 (35%)<br>CPS ≥1: N = 405 (65%)<br>CPS ≥ 10: N = 194 (31%)<br>CPS ≥ 20: N = 109 (18%)	CPS < 1: N = 211 (25%)<br>CPS ≥ 1: N = 636 (75%)<br>CPS ≥ 10: N = 323 (38%)	PD-L1 IC ≥ 1%: N = 368 (41%)<br>PD-L1 IC ≥1% and <5%: N = 243 (27%)<br>PD-L1 IC ≥ 5%:<br>N = 125 (14%)<br>Primary breast tumor samples<br>N = 563<br>PD-L1 ≥ 1%: N = 247 (44%)<br>Metastatic tumor samples N = 337<br>PD-L1 ≥ 1%: N = 121 (36%)	PD-L1 IC ≥ 1%: N = 292 (44.8%)<br>Primary breast tumor samples<br>N = 336<br>PD-L1 ≥ 1%: N = 161 (48%)<br>Metastatic tumor samples N = 315<br>PD-L1 ≥ 1%: N = 132 (42%)
**TILs population**	N = 110<br>Median IC level distribution 10% (range = 0.5-60%)	sTILs ≥ 5%: N = 39	Median sTILs level = 17.5% (range 6.2-57.5%)	N = 536<br>Median sTILs distribution 5% (IQR = 14%)	NR	N = 892<br>Median sTILs distribution 5% (IQR = 3%-10%; range = 0%-90%)	NR
**ORR**	All lines: 10%<br>Atezolizumab as first line therapy: 24%	20%	Cohort A: 5.3%<br>Cohort B : 21.4%	Pembrolizumab arm :<br>9.6%	Pembrolizumab arm: 40.8%<br>Placebo arm : 37.0%	Atezolizumab arm : 56%<br>Placebo arm : 45.9%	Atezolizumab arm : 54%<br>Placebo arm : 47%

	Atezolizumab as second line or beyond: 11%			Chemotherapy arm : 10.6%			
**mPFS**	1.4 months (95% CI 1.3-1.6)	1.9 months (95% CI 1.8-2.0)	Cohort A : 2.0 months<br>(95% CI 1.9-2.0)<br>Cohort B : 2.1 months<br>(95% CI 2.0-2.2)	Pembrolizumab arm :<br>2.1 months<br>Chemotherapy arm :<br>3.3 months<br>HR = 1.60 (95% CI 1.33-1.92)	Pembrolizumab arm :<br>7.5 months<br>Placebo arm : 5.6 months<br>HR = 0.82 (95% CI: 0.69-0.97)	Atezolizumab arm :<br>7.2 months<br>Placebo arm :<br>5.5 months<br>HR = 0.79 (95% CI 0.69-0.91)<br>P = 0.002	Atezolizumab arm :<br>5.7 months<br>Placebo arm :<br>5.6 months<br>HR = 0.86(95% CI 0.70-1.05)
**mOS**	8.9 months (95% CI 7.0-12.6)		Cohort A :<br>9.0 months (95% CI 7.6-11.2)<br>Cohort B :<br>18.0 months (95% CI 12.9-23.0)	Pembrolizumab arm :<br>9.9 months<br>Chemotherapy arm<br>10.8 months<br>HR = 0.97 (95% CI 0.82-1.15)	Pembrolizumab arm :<br>17.2 months<br>Placebo arm :<br>15.5 months<br>HR = 0.89 (95% CI 0.76-1.05)	Atezolizumab arm<br>21 months<br>Placebo arm<br>18.7 months<br>HR = 0.86 (95% CI 0.72-1.02)	Atezolizumab arm :<br>22.1 months<br>Placebo arm<br>28.3 months<br>HR = 1.11 (95% CI 0.76-1.64)
**SUBGROUPS ANALYSIS**							
**PD-L1**	IC 0/1 or IC 2/3	Responders or non-responders	Cohort A: CPS ≥ 1 or < 1	CPS ≥ 1, 10 or 20: Pembrolizumab vs chemo arm	CPS ≥ 1 or ≥10: Pembrolizumab vs placebo arm	IC ≥ 1% or < 1%: Atezolizumab vs placebo arm	IC ≥ 1%: Atezolizumab vs placebo arm
**ORR**	IC 0/1: ORR = 5% (95% CI 1.0-18.0)<br>IC 2/3: ORR = 12% (95% CI 6.0-22.0)	Responders: PD-L1 IC ≥ 1% = 15%<br>Non-responders: PD-L1 IC ≥ 1% = 5%<br>P = 0.02<br>Responders: PD-L1 TC ≥ 1% = 1%<br>Non-responders: PD-L1 TC ≥ 1% = 1%<br>P = 0.4	CPS ≥ 1: ORR = 5.7% (95% CI 2.4-12.2)<br>CPS < 1: ORR = 4.7% (95% CI 1.1-13.4)	CPS ≥ 1: ORR = 12.3 vs 9.4 %<br>CPS ≥ 10: ORR = 17.7 vs 9.2%<br>CPS ≥ 20: ORR = 26.3 vs 11.5 %	CPS ≥ 1: ORR = 44.9 vs 38.9%<br>CPS ≥ 10: ORR = 52.7 vs 40.8%	PD-L1 IC ≥ 1%: ORR = 58.9% vs 42.6%<br>HR = 1.96<br>95% CI 1.29-2.98	PD-L1 IC ≥ 1%: ORR = 63% vs 55%
**mPFS**	IC 0/1: mPFS = 1.4 months (95% CI 1.3-1.5)<br>IC 2/3: mPFS = 1.4 months (95% CI 1.3-1.9)<br>IC 1/2/3: mPFS = 1.4 months (95% CI 1.3-1.9)	NR	CPS ≥ 1: mPFS = 2.0 months (95% CI 1.9-2.1)<br>CPS < 1: mPFS = 1.9 months (95% CI 1.7-2.0)	CPS ≥ 1: mPFS = 2.1 vs 3.1 months<br>HR = 1.35 (95% CI 1.08-1.68)<br>CPS ≥ 10: mPFS = 2.1 vs 3.4 months	CPS <1: mPFS = 6.3 vs 6.2 months<br>HR = 1.08 (95% CI 0.77-1.53)<br>CPS ≥ 1: mPFS = 7.6 vs 5.6 months	PD-L1 IC ≥ 1%: mPFS = 7.6 vs 5.3 months<br>HR = 0.65 (95% CI 0.52-0.82)<br>PD-L1 IC <1%: mPFS = 5.6 vs 5.6 months	PD-L1 ≥ 1%: mPFS = 6 vs 5.7 months<br>HR = 0.82 (95% CI 0.60-1.12)

				HR = 1.14 (95% CI 0.82-1.59)<br>CPS ≥ 20: mPFS = 3.4 vs 2.4 months<br>HR = 0.76 (95% CI 0.49-1.18)	HR = 0.74 (95% CI 0.61-0.90)<br>CPS ≥ 10: mPFS = 9.7 vs 5.6 months<br>HR = 0.66 (95% CI 0.50-0.88)	HR = 0.92 (95% CI 0.77-1.10)	
**mOS**	IC 0/1: mOS = 7.0 months (95% CI 5.1-12.6)<br>IC 2/3: mOS = 10.5 months (95% CI 7.1-14.7)	NR	CPS ≥ 1: mOS = 8.8 months (95% CI 7.1-11.2)<br>CPS < 1: mOS = 9.7 months (95% CI 6.2-12.6)	CPS ≥ 1: mOS = 10.7 vs 10.2 months<br>HR 0.86 (95% CI 0.69-1.06)<br>P = 0.073<br>CPS ≥ 10: mOS = 12.7 vs 11.6 months<br>HR 0.78(95% CI 0.57-1.06)<br>P = 0.057<br>CPS ≥ 20: mOS = 14.9 vs 12.5 months<br>HR = 0.58 (95% CI 0.38-0.88) P = 0.50	CPS < 1: mOS = 16.2 vs 14.7 months<br>HR = 0.97 (95% CI 0.72-1.32)<br>CPS ≥ 1: mOS = 17.6 vs 16.0 months<br>HR = 0.86 (95% CI 0.72-1.04)<br>P= 0.0563<br>CPS ≥ 10: mOS = 23 vs 16.1 months<br>HR = 0.73 (95% CI 0.55-0.95)	PD-L1 IC ≥ 1%: mOS = 25.0 vs 18.0 months<br>HR = 0.71 (95% CI 0.54-0.94)<br>PD-L1 IC <1%: mOS = 19.7 vs 19.6 months<br>HR = 0.97 (95% CI 0.78-1.20)	PD-L1 ≥ 1%: mOS = 15.2 vs 15.8 months<br>HR = 1.11 (95% CI 0.76-1.64)
**TILs**	ICs level > 10% or ≤ 10%	Responders or non-responders	TILs ≥ or < median	TILs as continous variable	NR	sTIL ≥ 10% or < 10%: Atezolizumab vs placebo arm	NR
**ORR**	Median ICs level > 10%: ORR = 13% (95% CI 4.0-22.0)<br>Median ICs level ≤ 10%: ORR = 7% (95% CI 0-14.0)	Responders: sTILs = 22.5%<br>Non-responders: sTILs = 5%<br>P = 0.004	Cohort A :<br>Median Tils = 5%<br>Median sTILs 5%: ORR = 6%<br>Median sTILs < 5%: ORR = 2%<br>Cohort B:<br>Median sTILs = 17.5%<br>Median sTILs ≥ 17.5%: ORR = 39%<br>Median sTILs < 17.5%: ORR = 9%	Pembrolizumab arm ORR = 9.2%<br>P = 0.0007<br>Chemotherapy arm: ORR = 11%<br>P = 0.18	NR	NR	NR

**mPFS**	ICs level > 10% vs ≤ 10% HR = 0.76 (95% CI 0.51-1.14) P = 0.1855	NR	NR	NR		sTILs ≥ 10%: mPFS = 8.3 vs 6.1 months HR = 0.64 (95% CI 0.50-0.84) sTILs < 10%: mPFS = 5.6 vs 5.4 months HR = 0.86 (95% CI 0.73-1.02)	NR
**mOS**	ICs level > 10% vs ≤ 10% HR = 0.63 (95% CI 0.40-0.98) P = 0.0406	NR	NR	sTILs < 5% or ≥ 5%: Pembrolizumab vs chemo arm TILs < 5%: mOS = 5.9 vs 8.8 months TILs ≥ 5%: mOS = 12.5 vs 11.3 months	NR	sTILs ≥ 10%: mOS = 25 vs 20 months HR = 0.75 (95% CI 0.54-1.03) sTILs < 10%: mOS = 19.2 vs 18.1 months HR = 0.88 (95% CI 0.72-1.08)	NR
**CD8**	CD8+ ≥ 1.35% or < 1.35%	Responders or non-responders	NR	NR	NR	CD8+ ≥ 0.5% vs < 0.5%: Atezolizumab vs placebo arm	NR
**ORR**	CD8+ ≥ 1.35%: ORR = 14% (95% CI 4.0-23.0) CD8+ < 1.35%: ORR = 6% (95% CI 0-12.0)	Responders: CD8+ counts per mm² = 49.3 Non-responders: CD8+ counts per mm² = 25.5 P = 0.01	NR	NR	NR	NR	NR
**mPFS**	HR = 0.85 95% CI 0.55-1.31 P = 0.46	NR	NR	NR	NR	CD8+ ≥ 0.5%: mPFS = 7.4 vs 5.5 months HR = 0.75 (95% CI 0.62-0.91) CD8+ < 0.5%: mPFS = 5.6 vs 5.6 months HR = 0.86 (95% CI 0.65-1.14)	NR
**mOS**	HR = 0.68 (95% CI 0.41-1.12) P = 0.13	NR	NR	NR	NR	CD8+ ≥ 0.5%: mOS = 22.6 vs 18.1 months	NR

						HR = 0.69 (95% CI 0.54-0.87)<br>CD8+ < 0.5%: mOS = 16.3 vs 22.3 months<br>HR = 1.16 (95% CI 0.81-1.65)	
**TMB**	NR	Responders or non-responders	NR	Pembrolizumab or chemo arm	NR	NR	NR
**ORR**	NR	Responders: median TMB (mut/Mb) = 1.2<br>Non-responders: median TMB (mut/Mb) = 1.9<br>P = 0.1	NR	Pembro arm: ORR= 0.58 (95% CI 0.43-0.73) P = 0.154<br>Chemo arm: ORR=0.43 (95% CI 0.27-0.59) P=0.114	NR	NR	NR
**mPFS**	NR	NR	NR	NR	NR	PD-L1 positive or negative<br>PD-L1 positive: HR=0.31<br>95% CI 0.17-0.57<br>PD-L1 negative: HR=0.84<br>95% CI 0.48, 1.47	NR

TNBC Triple negative breast cancer ; IHC immunohistochemistry ; CPS Combined positive score ; TC Tumor cells ; IC Immune cells ; TILs Tumor infiltrating lymphocytes ; H&E Hematoxylin and eosin ; ORR Overall response rate ; mPFS median Progression free survival ; mOS median overall survival ; sTILs stroma TILs; TMB = tumor mutational burden, NR : not reported

Supplementary Table S2: Description of major clinical trials assessing ICIs as neoadjuvant treatment in TNBC with biomarkers assessment.

	**GeparNuevo**	**NeoTRIPaPDL1**	**Impassion 031**	**Keynote 522**
Study Design	Phase II<br>Neoadjuvant nab-paclitaxel and durvalumab vs nab-paclitaxel and placebo followed by adjuvant anthracycline and cyclophosphamide chemotherapy<br>Window cohort with one injection of durvalumab before combination therapy	Phase III<br>Neoadjuvant Carboplatin, nab-paclitaxel in association with atezolizumab vs placebo followed by adjuvant anthracycline based chemotherapy	Phase III<br>Neoadjuvant nab-paclitaxel and atezolizumab vs nab-paclitaxel and placebo followed by adjuvant anthracycline and cyclophosphamide	Phase III<br>Neoadjuvant paclitaxel and carboplatine in association with pembrolizumab vs placebo followed adjuvant anthracycline and cyclophosphamide
Population	N = 174<br>cT2 to cT4a-d	N = 280<br>All stage	N = 333<br>cT2–T4 and cN0–cN3	N = 1174<br>T1cN1-2 or T2-4N0-2
Biopsy requirement for biomarker assessment	Pre-treatment biopsy<br>Post-treatment biopsies after durvalumab in window cohort	Pre-treatment, on-treatment (day 1, cycle 2) biopsy and surgery	Pre-treatment biopsy and surgery	Pre-treatment biopsy and surgery
PD-L1 testing and scoring	IHC Ventana SP263<br>Positive if PD-L1 TC or IC ≥ 1%	IHC Ventana SP142<br>PD-L1 scored on IC and TC:<br>IC0 <1%<br>IC1 ≥1% and <5%<br>IC2 ≥ 5% and <10%<br>IC3 ≥ 10%	IHC Ventana SP142<br>Positive if PD-L1 IC ≥ 1%	IHC 22C3<br>Positive if CPS ≥ 1
PD-L1 population	PD-L1 positive: N = 138 (87%)	IC1: N = 91 (32.5%)<br>IC2: N = 48 (17%)	PD-L1 positive: N = 152 (46%)	PD-L1 positive: N = 973 (83%)

TILs population	sTILs 0-10%: N = 66 (38%)<br>sTILs 11-59%: N = 83 (48%)<br>sTILs ≥ 60%: N = 25 (14%)	sTILs ≥ 40%: N = 59 (34%)		
pCR rate	Durvalumab vs Placebo<br>Whole cohort<br>53.4 vs 44.2 %<br>OR = 1.45 (95% CI 0.80–2.63)<br>P = 0.224<br>Window cohort<br>61 % vs 41.4 %<br>OR = 2.22 (95% CI 1.06–4.64)<br>P = 0.035<br>No window cohort<br>37.9% vs 50%<br>OR = 0.61 (95% CI 0.21–4.17)<br>P = 0.360	Atezolizumab vs Placebo<br>52.3 vs 47.7 %<br>P = 0.46	Atezolizumab vs Placebo<br>58% vs 41%<br>P = 0.0044	Pembrolizumab vs Placebo<br>64.8% vs 51.2% (95% CI 5.4 - 21.8)<br>P < 0.001
mDFS	NR	NR	HR = 0.74<br>95% CI 0.32-1.70<br>P = NS	DFS at 18 months: 91.3% vs 85.3%<br>HR = 0.63<br>95% CI 0.43 - 0.93
mOS	NR	NR	HR = 0.69<br>95% CI 0.25-1.87<br>P = NS	
**pCR, SUBGROUPS ANALYSIS**				

PD-L1	Durvalumab arm: PD-L1 positive vs negative<br>58% vs 44.4%<br>OR = 1.72 (95% CI 0.43-6.98) P = 0.445<br>Placebo arm: PD-L1 positive vs negative<br>50.7% vs 18.2 %<br>OR = 4.63 (95% CI 0.93-23.01) P = 0.061	Atezolizumab vs Placebo arm<br>IC0 : 35.1% vs 41.1%<br>IC1 : 56,2% vs 44%<br>IC2/3 : 87% vs 72%	PD-L1 positive: Atezolizumab vs Placebo arm<br>69% vs 49%<br>P = 0.021<br>PD-L1 negative: Atezolizumab vs Placebo arm<br>48% vs 34%<br>P = NS	PD-L1 positive : Pembrolizumab vs Placebo arm<br>68.9% vs 54.9%<br>PD-L1 negative: Pembrolizumab vs Placebo arm<br>45.3% vs 30.3%
TILs	Durvalumab arm<br>Baseline sTILs as continuous marker<br>OR = 1.23 (95% CI 1.04-1.6) P = 0.019<br>Baseline iTILs as continuous marker<br>OR = 1.58 (95% CI 0.85-2.97) P = 0.150<br><br>Placebo arm<br>Baseline sTILs as continuous marker<br>OR = 1.39 (95% CI 1.12-1.74) P = 0.003<br>Baseline iTILs as continuous marker<br>OR = 0.94 (95% CI 0.73-1.22) P = 0.636	Atezolizumab arm<br>sTIls high vs low<br>OR = 3.41 (95% CI 1.32-8.82) P = 0.01<br>sTILs Intermediate vs low<br>OR = 1.62 (95% CI 0.71-3.74) P = 0.25<br>iTILs high vs low<br>OR = 6.84 (95% CI: 2.60-18.01) P = 0.0001<br>iTILs intermediate vs low<br>OR = 2.66 (95% CI: 1.13-6.30) P = 0.03<br><br>Placebo arm<br>sTIls high vs low OR = 1.76 (95% CI 0.75-4.17) P = 0.19	NR	NR

		sTILs Intermediate vs low OR = 0.63 (95% CI 0.26-1.55) P = 0.3<br>iTILs high vs low OR = 2.22 (95% CI 0.95-5.22) P = 0.06<br>iTILs intermediate vs low<br>OR = 1.44 (95% CI 0.59-3.49) P = 0.3		

IHC immunohistochemistry ; TC Tumor cells ; IC Immune cells ; CPS Combined positive score ; TILs Tumor infiltrating lymphocytes ; H&E Hematoxylin and eosin ; pCR pathologic complete response ; OR Odd Ratio ; mDFS median Disease free survival ; NS not significant ; mOS median overall survival ; sTILs stroma TILs ; iTILS intra-tumor TILs ;
